# Supplementary material for: A synergistic generative-ranking framework for tailored design of therapeutic single-domain antibodies
Source: Cell Discov. 2025 Oct 29;11:85. doi: 10.1038/s41421-025-00843-8 (PMC12572175; doi:10.1038/s41421-025-00843-8)
Supplement: Supplementary file 1 — Supplementary Information [file 41421_2025_843_MOESM1_ESM.pdf]

Supplementary Materials for

A synergistic generative-ranking framework for tailored design of therapeutic single-domain antibodies

Yu Kong[1#], Jiale Shi[1#], Fandi Wu[2#], Ting Zhao[1#], Rubo Wang[2,5#], Xiaoyi Zhu[1], Qingyuan Xu[3], Yidong Song[2,6], Quanxiao Li[1], Yulu Wang[1], Xingyu Gao[7], Yuedong Yang[8], Yi Feng[1], Zifei Wang[1], Weifeng Ge[9], Yanling Wu[1,4*], Zhenlin Yang[3,4*], Jianhua Yao[2*], Tianlei Ying[1,4*]

[*]Correspondence: yanlingwu@fudan.edu.cn (Y.W.), yang_zhenlin@fudan.edu.cn (Z.Y.), jianhuayao@tencent.com (J.Y.), tlying@fudan.edu.cn (T.Y.)

List of Supplementary Materials

Supplementary Fig. Sl to Supplementary Fig. S6

Table S1 to Tables S2

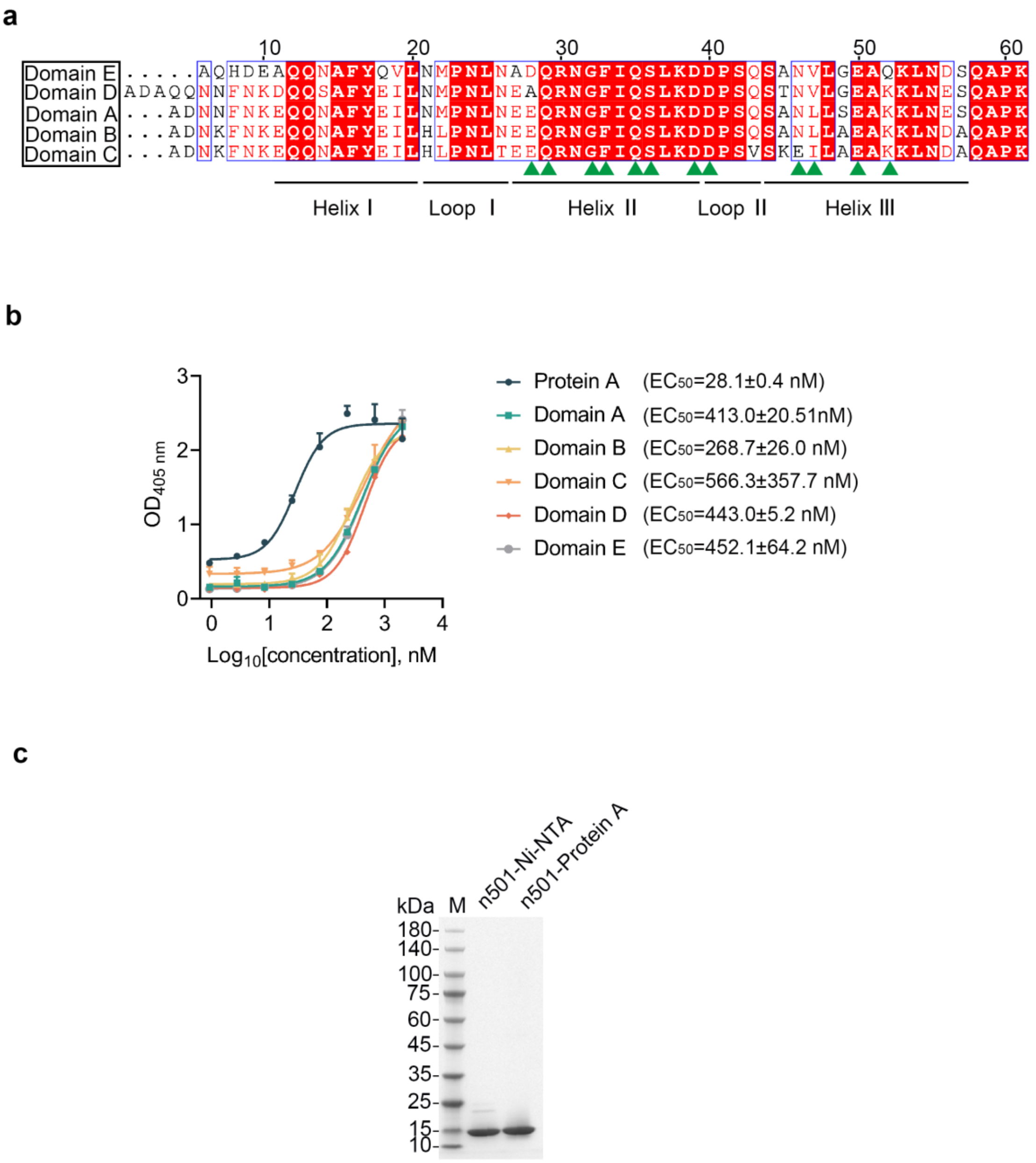

Supplementary Figure S1: sdAb n501 can be purified using Protein A resin. a. Amino acid sequence alignment of the five domains of Protein A. Loops and helix regions in the structure are marked, with green arrows indicating the amino acids in

domain D that are involved in binding with n501. **b.** ELISA assay results for the binding of n501 with Protein A and its various domains. The EC50 values are also presented. The experiment was conducted with three replicates. **c.** SDS-PAGE analysis of the purification products of n501 using Ni-NTA and Protein A resins.

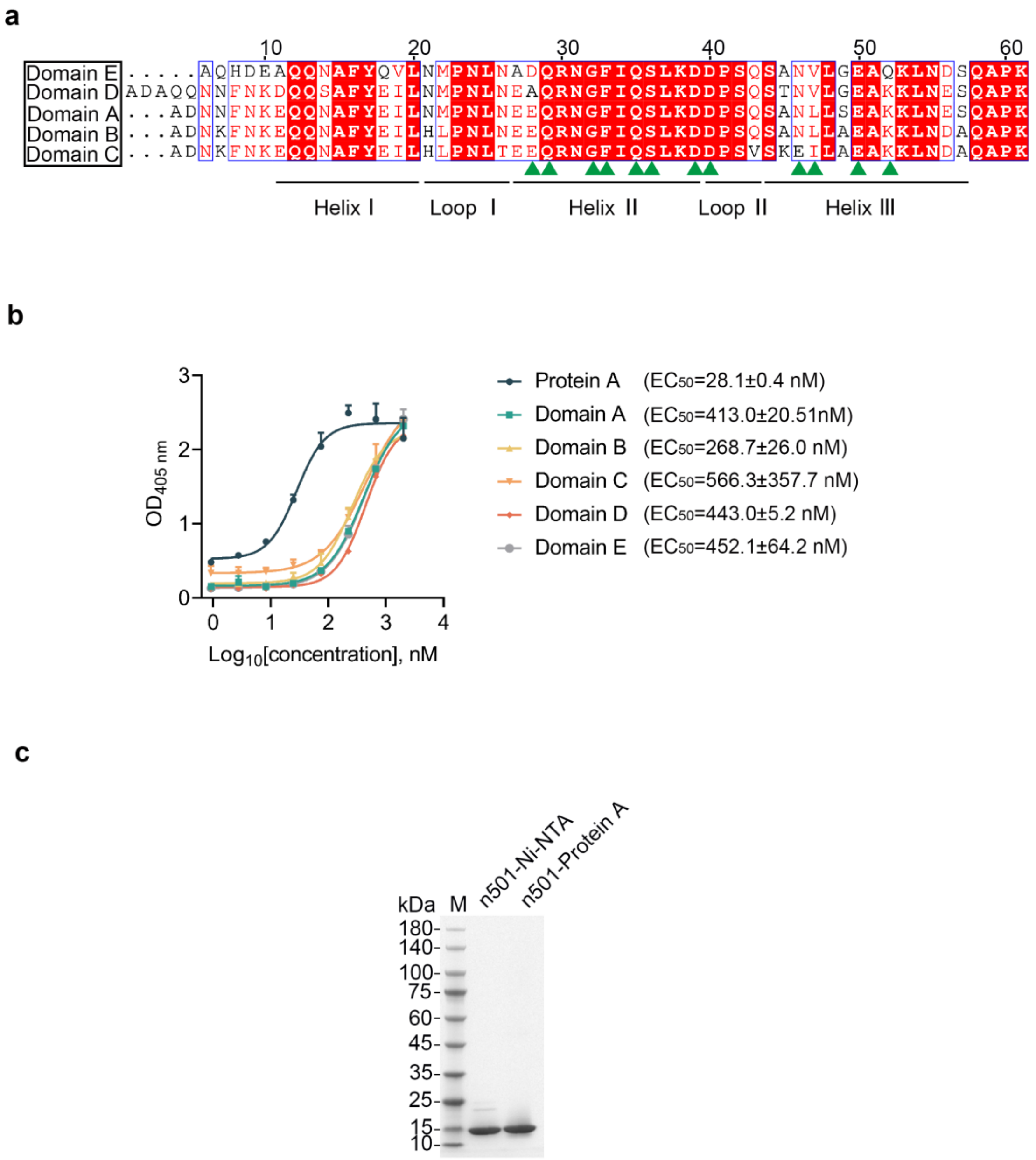

Supplementary Figure S2: The framework region is primarily responsible for the binding of n501 to Protein A. a. Sequence alignment of n501 with two chimeric sdAbs where the CDRs are grafted onto the framework regions of n501. Identical

amino acids are represented by dots. The three CDRs are individually marked. **b.** BLI binding assay for two chimeric sdAbs and Protein A. **c.** The structural comparison between n501-domain D and n501-5T4 (PDB) is presented. n501, domain D, and 5T4 are represented in palegreen, lightblue, and grey, respectively, with CDR1 and CDR2 indicated in orange and CDR3 in red.

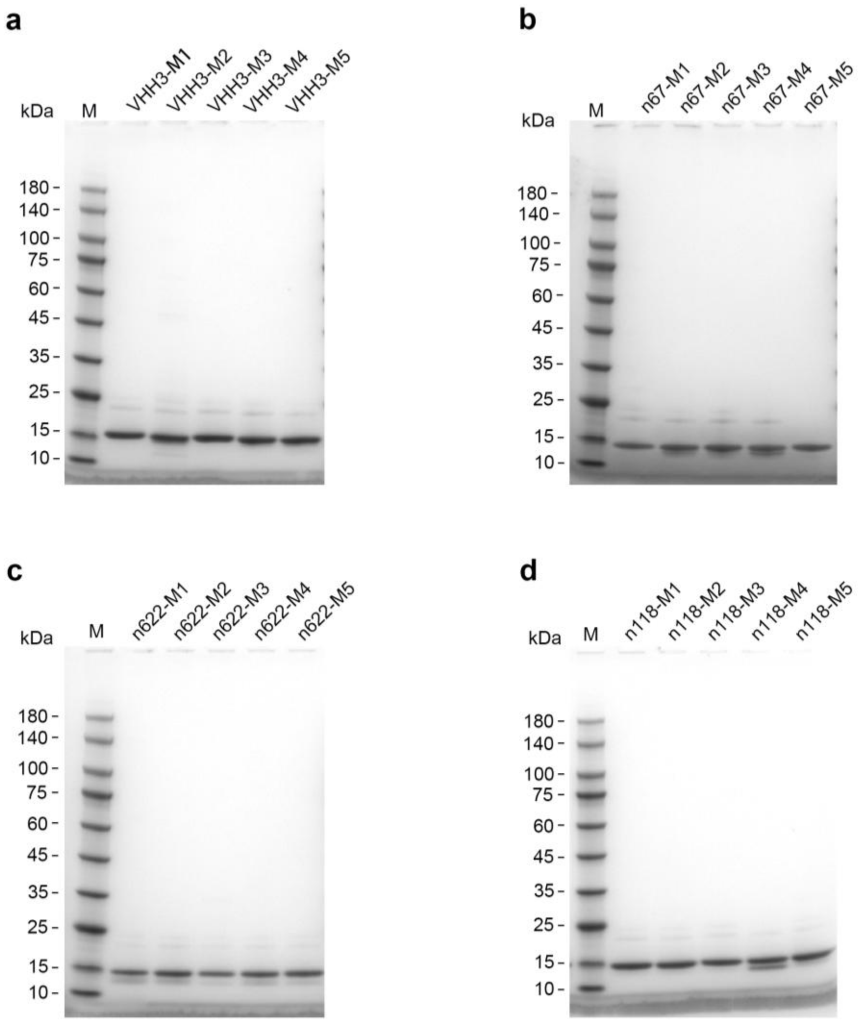

Supplementary Figure S3: SDS-PAGE analysis of sdAb mutants. a. Camelid-derived nanobody VHH3 variants. **b-d.** The mutants of human sdAbs including n67, n622, and n118.

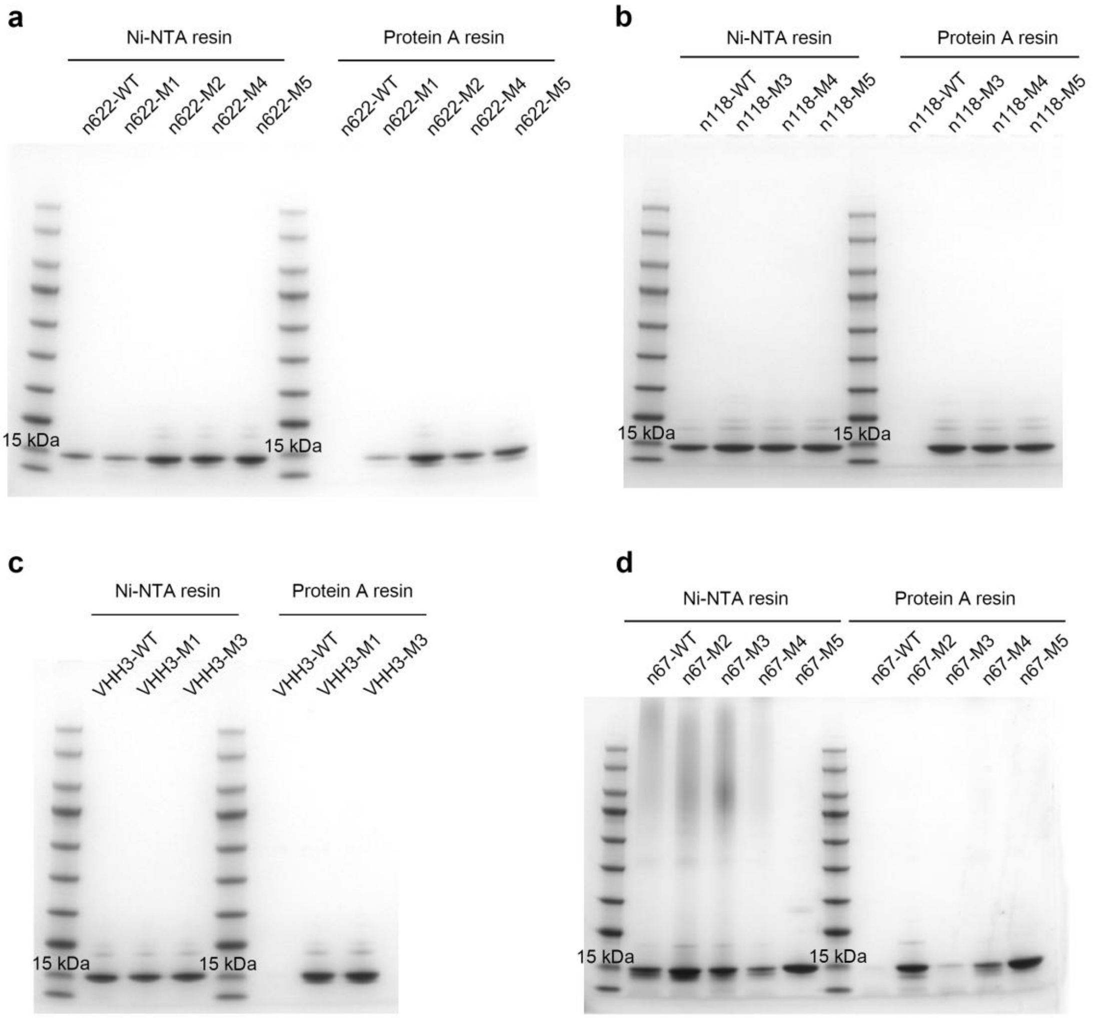

Supplementary Figure S4: SDS-PAGE analysis was conducted to evaluate the purification efficiency of sdAb mutants by Ni-NTA and Protein A resins. a. Camelid-derived nanobody VHH3 variants. **b-d.** The mutants of human sdAbs including n67, n622, and n118.

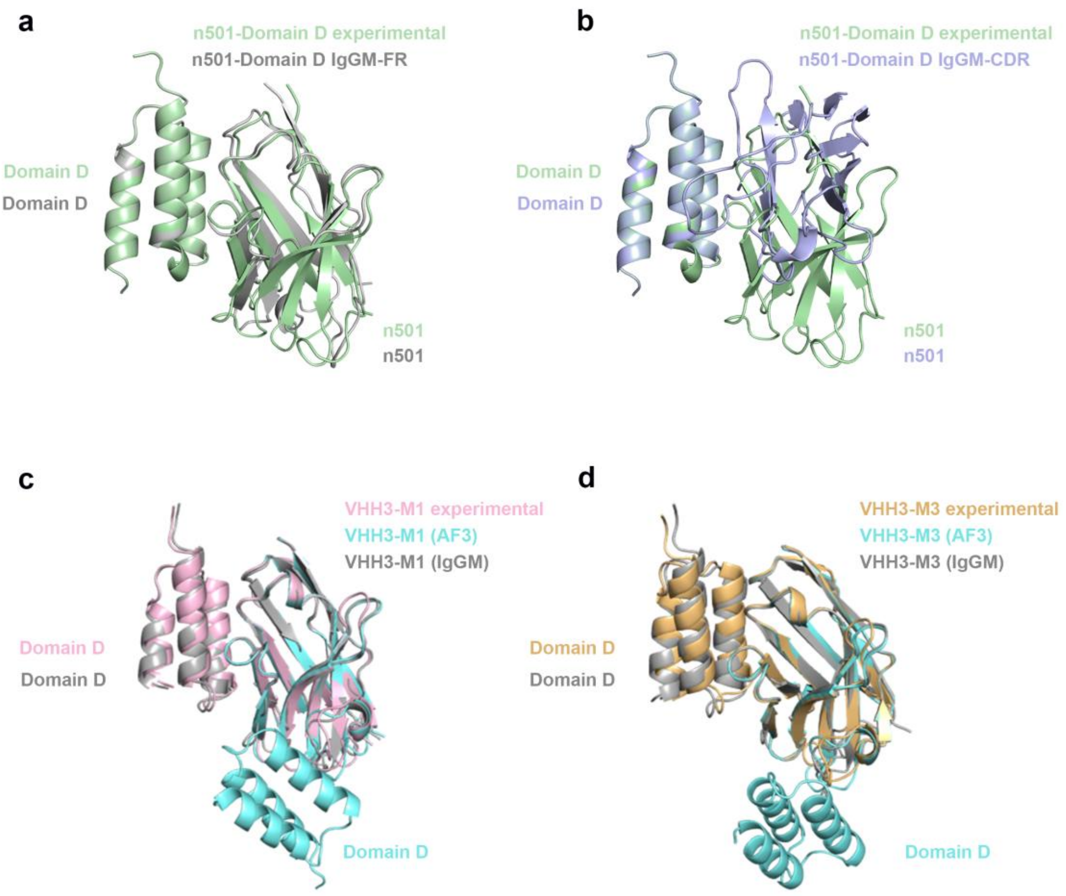

Supplementary Figure S5: Evaluation of structure prediction. a. Alignment of the n501-Domain D complex structure using the IgGM-FR (grey) and X-ray (green). **b.** Alignment of the n501-Domain D complex structure using the IgGM-CDR (light blue) and X-ray (green). **c-d.** Comparison of the structural prediction of IgGM with that of AlphaFold-3 for the VHH3-M1-Protein A (c) and VHH3-M3-Protein A (d) complexes.

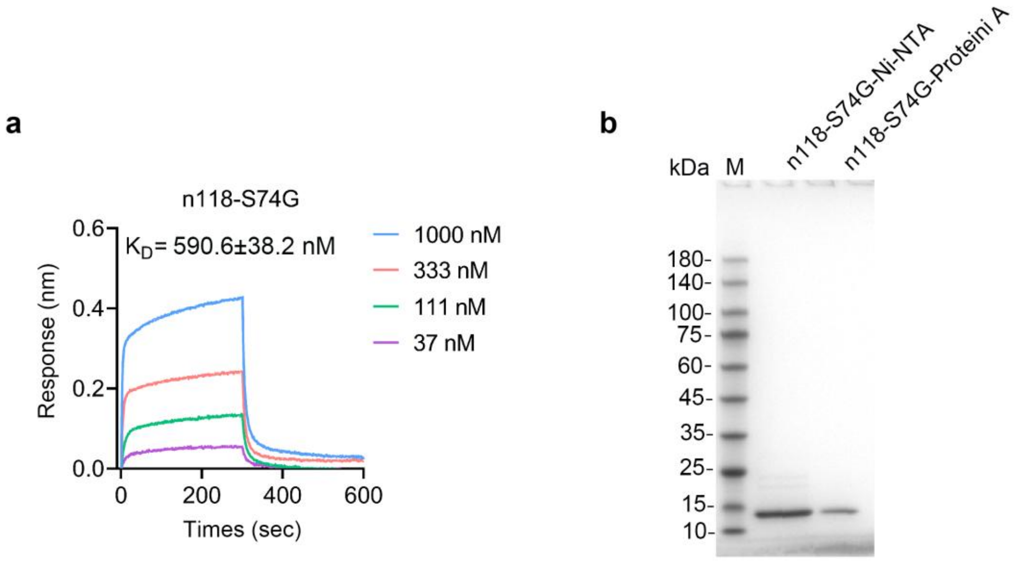

Supplementary Figure S6: n118-S74G bind to Protein A. a. BLI binding assay for n118-S74G and Protein A. **b.** SDS-PAGE analysis of the purification products of n118-S74G using Ni-NTA and Protein A resins.

Supplementary Table 1. Interactions between n501/VHH3-M1/M3 and Domain D

n501 location	Domain D residues	Helix II	Loop	Helix III
	G16	Q29 (H)		
FR1	S18	A28 (H)		
	R20	Q35, D39(S)		
	T65		D40(H)	
	Y67		D40(H)	
	K72			N46
	G74			N46, V47, E50
FR3	T77	D39, S36(H)		
	S79	D39		
	Q90	Q35, S36		
	N92	G32(H), F33, S36(H)		
	R95			E50(S), K53

VHH3-M1/M3 location	Domain D residues	Helix II	Loop	Helix III
	G16	Q29(H)		
FR1	S18	A28		
	R20	Q35, D39(S)		
	T65		D40(H), S42	
	Y67		D40(H), Q43	
	K72			E50
	G74	F33		N46, V47, E50
FR3	T77	S36(H), D39(H)	D40	
	S79	D39		
	Q90	G32, Q35, S36, D39,		
	N92	G32(H), F33, S36（H）		
	S93			L54
	R95			E50(S)

H: Hydrogen bond; S: Salt bridge

Supplementary Table S2. X-ray crystallography data collection and refinement statistics

**Data collection**	**n501**	**VHH3-M1**	**VHH3-M3**
**PDB ID**	**9M5D**	**9M6O**	**9M6J**
Wave length (Å)	0.97903	0.9792	0.9792
Space group	*I422*	*P212121*	*C2221*
Cell dimensions			
a, b, c (Å)	173.97, 173.97, 79.61	52.93, 78.48, 97.17	78.58, 98.12, 52.65
α, β, γ (°)	90.0, 90.0, 90.0	90.0, 90.0, 90.0	90.0, 90.0, 90.0
Resolution (Å)	55.02-3.57(3.698-3.57)	29.94-1.49(1.51-1.49)	25.31-2.0(2.15-2.0)
Mean I/sigma(I)	4.12(1.83)	7.73(1.16)	18.14(3.06)
Completeness (%)	99.67(99.71)	99.55(98.92)	98.50(96.90)
Multiplicity	1.1(1.1)	1.0(1.0)	1.0(1.0)
**Refinement**			
No. reflections	7,511(2433)	66,537(2,843)	13,892(2,684)
R_{work} / R_{free} (%)	23.96/27.62	22.44/25.66	19.24 / 22.57
Number of non-hydrogen atoms	2,714	3,151	1,539
Macromolecules	2,714	2,800	1,386
Ligands	0	0	0
Solvent	0	351	153
Average B-factor (Å^2)	76.08	20.32	22.18
Macromolecules	76.08	19.26	21.06
Ligands	/	/	/
Solvent	/	28.78	32.32
R.m.s. deviations	/	/	/
Bond lengths (Å)	0.004	0.006	0.006
Bond angles (°)	0.78	0.89	0.77
Ramachandran			
Favored (%)	98.53	99.15	98.28
Allowed (%)	1.47	0.85	1.72
Outliers (%)	0.0	0	0.0
